# Inter-Identity Autobiographical Amnesia in Patients with Dissociative Identity Disorder

**DOI:** 10.1371/journal.pone.0040580

**Published:** 2012-07-18

**Authors:** Rafaële J. C. Huntjens, Bruno Verschuere, Richard J. McNally

**Affiliations:** 1 Department of Clinical Psychology, University of Groningen, Groningen, The Netherlands; 2 Department of Clinical Psychology, University of Amsterdam, Amsterdam, The Netherlands; 3 Department of Psychology, Ghent University, Ghent, Belgium; 4 Faculty of Psychology and Neuroscience, Maastricht University, Maastricht, The Netherlands; 5 Department of Psychology, Harvard University, Cambridge, Massachusetts, United States of America; The University of Queensland, Australia

## Abstract

**Background:**

A major symptom of Dissociative Identity Disorder (DID; formerly Multiple Personality Disorder) is dissociative amnesia, the inability to recall important personal information. Only two case studies have directly addressed autobiographical memory in DID. Both provided evidence suggestive of dissociative amnesia. The aim of the current study was to objectively assess transfer of autobiographical information between identities in a larger sample of DID patients.

**Methods:**

Using a concealed information task, we assessed recognition of autobiographical details in an amnesic identity. Eleven DID patients, 27 normal controls, and 23 controls simulating DID participated. Controls and simulators were matched to patients on age, education level, and type of autobiographical memory tested.

**Findings:**

Although patients subjectively reported amnesia for the autobiographical details included in the task, the results indicated transfer of information between identities.

**Conclusion:**

The results call for a revision of the DID definition. The amnesia criterion should be modified to emphasize its subjective nature.

## Introduction

A major symptom of Dissociative Identity Disorder (DID; formerly Multiple Personality Disorder) is dissociative amnesia, an inability to recall important personal information that is too extensive to be explained by ordinary forgetfulness [1]. Spiegel et al. [2] suggested that the DSM-V committee broaden the amnesia criterion to include the inability to recall *everyday events* as well as the inability to recall important personal information (e.g., traumatic events**)**.

The DID patient’s inability to recall information presumably arises from the compartmentalization of memories in separate identity states [3]. In experimental research, compartmentalization is assessed by one identity learning new information and another identity, reporting amnesia for the learning trial, being tested on retrieval of this information. The majority of compartmentalization studies in DID included neutral stimulus material, usually unrelated words or drawings of common objects [4]–[14], whereas only a few studies have also included emotionally valenced stimulus material [15]–[17]. One example of a controlled study concerning neutral stimulus material is an interference paradigm [11], [14]. First, we asked participants to learn a list of words containing trauma-related, positive, and neutral words (list A). We then tested the patients for wordlist free recall. Subsequently, and following a switch to an amnesic identity, we asked the patients to learn a wordlist B containing different words from the same semantic categories, again followed by a free recall test. In contrast to a hypothesis of inter-identity amnesia, the DID participants *recalled* words from List A in their amnesic identity (i.e., the identity learning List B), indicating transfer of newly learned material between identities. Additionally, after a two-hour interval, the amnesic identity (i.e., exposed to list B) performed a surprise recognition test. We showed this identity all the words from both lists intermixed with distractor words (i.e., new words from the same semantic categories) and asked it to indicate which words were old (i.e., seen in the learning phase) and which were new. Again, inconsistent with the hypothesis of interidentity amnesia, participants recognized List A words in their amnesic dissociative identity. These results were replicated and extended in a different patient group by Kong et al. [14] who included a cross-modal manipulation designed to mitigate implicit memory effects. Furthermore, Huntjens et al. [17] used negatively and positively valenced words to test whether the presumptive amnesic barrier is especially impermeable to negative material, as implied by the belief that amnesia in DID functions to block painful memories. Consistent with previous studies, transfer between identities on the memory task occurred even for negative material, despite patients reporting amnesia for this material, learned in another identity state. Transfer across amnesic barriers in DID also occurs for conditioned emotional information. Testing DID participants, Huntjens et al. [16] administered an evaluative conditioning procedure that confers a positive or negative connotation on neutral words. In a subsequent affective priming procedure, participants displayed transfer of this newly acquired emotional valence to the amnesic identity (i.e., transfer of emotional material between identities).

The results of these and other controlled studies [18], [19] indicate intact interidentity memory functioning in DID even though patients subjectively report experiencing amnesia between identities. Memory transfer occurs for both implicit and explicit memory retrieval tasks, and for both neutral and emotional material. Nevertheless, DID experts hold that a deficit in both episodic and semantic aspects of *autobiographical memory* (i.e., dense amnesia for personal identity and a substantial portion of one’s life history) to be the central phenomenon of dissociative amnesia [2]. However, in the experiments described above, the patients did not generate the stimulus materials, nor did they rate the material for personal relevance. Indicating that autobiographical memory might be a prerequisite for compartmentalization, two case studies directly addressed autobiographical memory performance in DID, and both provided evidence for autobiographical amnesia [20], [21]. To illustrate, Bryant examined a 31-year old DID patient in two conditions: As the predominant (“host”) identity of the DID patient, and as a nine-year-old trauma identity claiming awareness of abuse of which the host identity was unaware. Importantly, the host identity did not report abuse memories, whereas the child identity reported no memories of the recent past. Although the data from the two case studies suggest autobiographical amnesia in DID, they have important shortcomings. First, that one identity does not report certain memories does not necessarily mean that these memories are truly inaccessible. Failing to mention a memory does not necessarily mean that the person is *unable* to recall it [22]. True amnesia would entail an inability to recall these memories, whereas unwillingness might suggest malingering or factitious behavior. Second, each study examined only a single DID patient.

**Figure 1:**
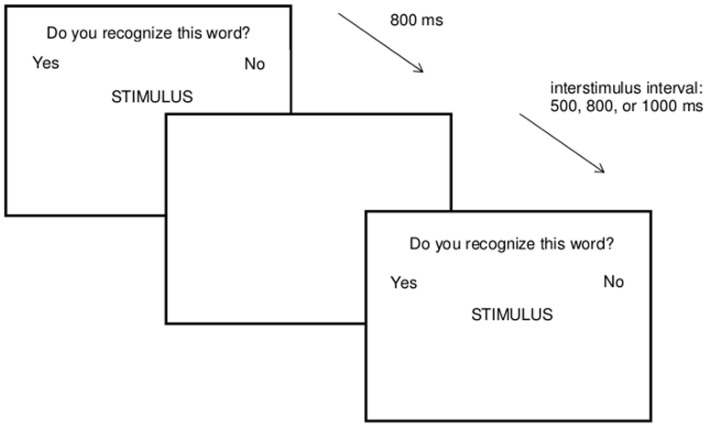
Procedure concealed information task.

The purpose of the present study was to use an objective memory measure - the concealed information test - to assess recognition of autobiographical information in DID patients across identities [23], [24]. Using the concealed information test, Allen and Movius [9] found transfer in DID for neutral stimuli. Here, we applied the concealed information test for the assessment of autobiographical information. If patients exhibit inter-identity amnesia, then their reaction times to classify other-identity items (i.e., autobiographical information from the identity for which the tested identity reports amnesia) should be indistinguishable from their reaction times to comparable, yet irrelevant items. If they exhibit memory transfer between identities, then their reaction times to classify other-identity items should be longer than to classify irrelevant items, implying their recognition of the former as self-relevant. We also tested matched controls, and asked simulators to feign amnesia for the self-generated other-identity items. The latter group was included to control for conscious malingering of amnesia. The inter-identity amnesia hypothesis predicts a null finding. To allow meaningful interpretation of such a finding, we also included “same identity” items, as a benchmark. For these items, we expected longer reaction times compared to the irrelevant items in all groups, thereby demonstrating the test’s sensitivity.

**Table 1:** Subject demographics.

	DID(n = 9)	Controls(n = 27)	Simulators(n = 23)
Age, Mean Years(SD)	43.67 (11.21)	41.30 (12.95)	41.00 (14.18)
Education, M (SD)	5.11 (1.69)	6.00 (0.68)	5.87 (1.06)
DES, M (SD)	46.47 (13.66)	7.10 (4.98)	5.65 (5.03)
SDQ, M (SD)	10.78 (2.86)	5.37 (0.79)	5.65 (1.03)

*Note*. Education was assessed on a scale from 1 (low) to high (7) [34].

## Methods

### Participants and Ethics Statement

Eleven female DID patients participated as did 27 healthy female control subjects and 24 DID simulating female control subjects. We recruited DID patients from treatment settings in the Netherlands and Belgium by asking clinicians to invite patients to participate. The clinician’s diagnosis of DID was verified with the Dutch version of the Structured Clinical Interview for *DSM-IV* Dissociative Disorders (SCID-D) [25] by the first author. Validating the Dutch version, Boon and Draijer [26] reported an excellent interrater reliability for presence versus absence of a dissociative disorder (κ = .96) and for type of dissociative disorder (κ = .70).

**Table 2:** Mean (SD) stimulus rating for personal relevance.

	DID(n = 9)	Controls(n = 27)	Simulatorsn = 23)
Target items	3.22 (1.78)	2.25 (1.27)	2.80 (1.74)
Irrelevant items	2.64 (1.53)	1.30 (0.36)	1.54 (0.57)
Other identity items	3.67 (2.14)	1.14 (0.21)	1.58 (0.59)
Same identity items	7.56 (1.01)	7.57 (1.22)	8.32 (0.87)

DID was always the main reason for patients to be in treatment; all had a history of multiple hospitalizations and a relatively chronic course. The mean length of treatment for DID was 8.90 years (SD = 5.89). The mean number of identities reported by patients was 12.80 (SD = 11.96; range 4–39). Patients self-selected two identities for participation in the experiment, with one identity reporting awareness of a traumatic past (called the trauma identity) and the other identity reporting no memories of the traumatic past (called the amnesic identity). Furthermore, the selection of identities was based on: (1) the ability to switch between identities on request; (2) the ability to perform the tasks without spontaneous switches to or interference from other identities; (3) the ability to read and write, and (4) sufficient stability to perform computer tasks.

**Table 3:** Mean (SD) proportion error scores on target, irrelevant, other identity and same identity items for DID patients, controls and simulators.

	DID(n = 9)	Controls(n = 27)	Simulators(n = 23)
Target items	.32 (.17)	.19 (.09)	.21 (.12)
Irrelevant items	.05 (.04)	.02 (.02)	.02 (.02)
Other identity items	.02 (.02)	.02 (.03)	.08 (.13)
Same identity items	.10 (.13)	.04 (.07)	.24 (.28)

We included twenty-seven female control subjects matched on age and education level. They were community volunteers. Additionally, we included 24 female amateur actors in a simulation group and asked to mimic DID. Their mean years of theater experience was 13.78 years (SD = 11.51; range 1–45 years). We showed them a documentary film about a DID patient and gave them additional written information about DID. Subsequently, we asked them to create two imaginary identities. One identity had to have memories of personally experienced childhood sexual abuse, whereas the other was to be amnesic for the abuse. Following the procedure of previous studies on DID [5], [11], simulators received a data sheet for the identity on which we asked them to assign a name, age, gender, physical description, personal history, and personality style of the identities. Finally, we asked them to practice switching their identities during the week preceding their participation in the experiment.

We excluded control and simulator participants who reported any relevant memory, visual, or attentional problems. We used the Mini-International Neuropsychiatric Interview (M.I.N.I.) [27] to ensure that healthy control subjects had no current psychiatric disorder, and we excluded potential control subjects with a history of sexual or physical abuse. All participants completed the Dissociative Experiences Scale (DES) [28]. The DES is a 28-item self-report questionnaire with scores ranging from 0 to 100. Scores above 20 or more conservatively, above 30 suggest pathological dissociation. In the present study, the DES demonstrated good internal consistency (Cronbach’s alpha = .97). To measure somatoform dissociation, we included the Somatoform Dissociation Questionnaire (SDQ-5). The SDQ-5 is a shortened version of a 20-item questionnaire that assesses somatic symptoms associated with dissociation such as motoric inhibition, intermittent pain symptoms, and anesthesia [29]. The authors of the SDQ-5 say that a score greater than 7 discriminates dissociative from other disorders. In the present study, the SDQ-5 demonstrated modest internal consistency (Cronbach’s alpha = .63).

We informed DID patients that the aim of the study is to investigate the memory problems reported by patients with DID. We did not explain to subjects in the normal control group that their scores would be compared to patients with DID. All subjects provided written informed consent prior to participating, and all received payment of 50 Euros. The study was approved by the Medical Ethical Committee of the University Medical Centre Groningen, The Netherlands.

**Table 4:** Mean reaction times (SD) for target, irrelevant, other identity and same identity items for DID patients, controls and simulators.

	DID(n = 9)	Controls(n = 27)	Simulators(n = 23)
Target items	590.44 (44.29)	564.69 (48.59)	572.11 (40.57)
Irrelevant items	461.50 (57.90)	448.78 (40.01)	470.67 (50.37)
Other identity items	487.39 (57.42)	451.91 (40.23)	496.54 (64.71)
Same identity items	491.06 (59.76)	480.41 (42.93)	525.11 (82.14)

**Figure 2:**
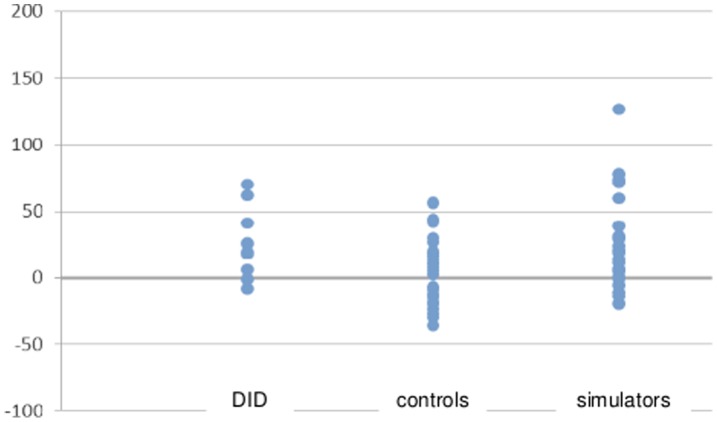
Individual difference scores for other identity and irrelevant scores for DID patients, control participants, and simulators.

**Figure 3:**
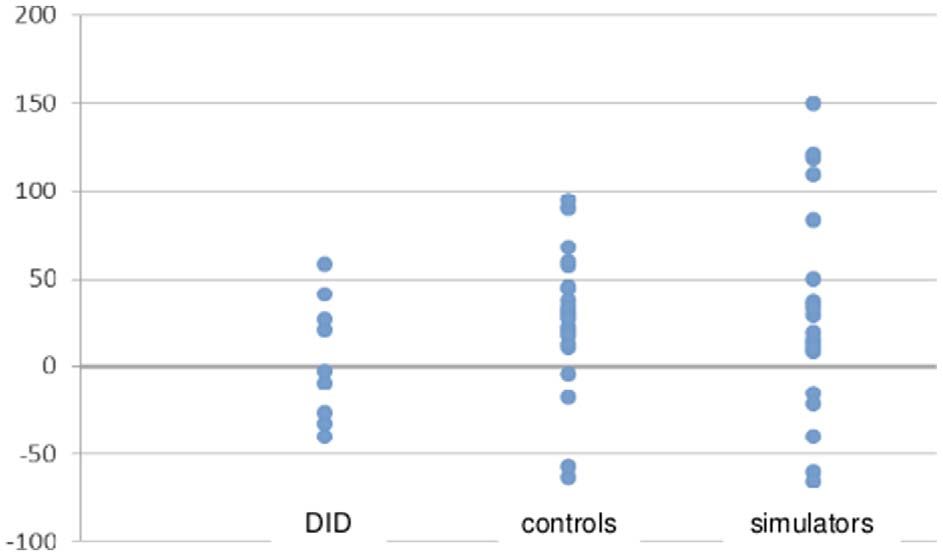
Individual difference scores for same vs. other identity scores for DID patients, control participants, and simulators.

**Figure 4:**
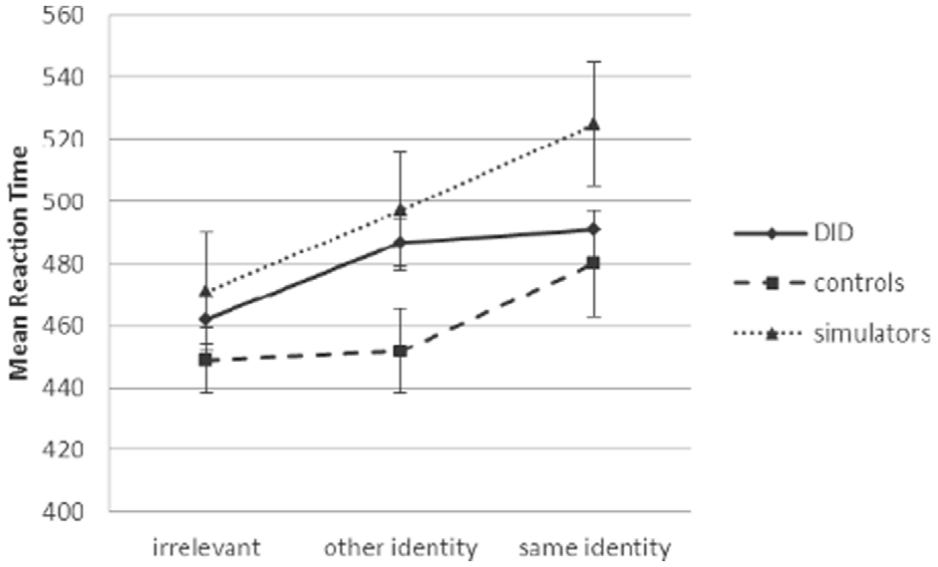
Mean reaction time for same identity, other identity, and irrelevant items for DID patients, control participants, and simulators.

### Materials and Procedure

In session 1, the patients provided basic demographic information and completed 20 autobiographical information questions (e.g., name, date and place of birth, names of sisters, brothers, partners, children, and best friends, name of primary school, favorite sport and hobby, movie that made a lasting impression, favorite band or composer, favorite holiday destination, and favorite food). The patients completed the questionnaire twice in the laboratory. First, the trauma identity was asked to complete the autobiographical questionnaire. Then, we asked patients to switch to the amnesic identity state, and asked to complete the questionnaire again. Finally, the amnesic identity was also asked to complete the questions for the trauma identity. It was stressed that they were asked to answer the questions themselves without help of other identities and that they were allowed to leave it blank if they did not know the answer. In session 1, patients also completed the diagnostic interview (SCID-D) and several questionnaires. The healthy controls and simulators completed the questionnaires at home as well as a telephone diagnostic interview (M.I.N.I.).

In session 2, one week later, all participants came to the lab. They rated all stimuli of the concealed information task on personal relevance on a scale from 1 (not personally relevant) to 9 (very personally relevant). Patients completed the rating scale in their amnesic identity. Controls filled in the rating scale as themselves and we instructed the simulators that the amnesic identity was amnesic for the information pertaining to the trauma identity.

The critical concealed information task was performed in the third session, one week after the second session. We selected between two to four control participants based on their mean age, level of education, and questionnaire answering, to match an individual DID patient on the three autobiographical information categories included in the concealed information task. The task was described to participants as a memory study and they first learned a set of three target stimuli, consisting of one preselected word for each of the three selected autobiographical information categories (e.g., FAVORITE FOOD: pancakes; HOBBY: painting; FAVORITE MUSIC: Queen). Each word was shown for 30 s on a computer screen and after presentation of all the words, memory for the target items was assessed by asking the participants to type in each target word (e.g., FAVORITE FOOD?). This procedure was repeated two more times to perfect performance. In the subsequent critical phase, we asked participants to classify several stimuli in two categories: target versus non-target. The crucial manipulation was that the autobiographical same identity and (for patients) other identity items were embedded within the non-target category, and participants had to classify these stimuli as non-target items. The response to these autobiographical items was contrasted with the response to irrelevant control items. The target items and irrelevant control items were chosen prior to the task. They were checked beforehand and changed if necessary to ensure that they did not resemble any of the autobiographical items. Also, they were chosen to match the autobiographical items in mean number of letters. For normal control subjects, the other identity items consisted of irrelevant items comparable to the other irrelevant non-target items in mean number of letters. Overall, the mean number of letters for stimulus words was 8.56 (SD = 3.79). There were no significant differences between different word categories in mean number of stimulus letters per word and no significant differences between the groups for the different word categories.

In the concealed information task (see Figure 1), we presented participants with one item (target, other identity, same identity, or irrelevant) on each trial. They were to respond with the yes-button by using one hand and with the no-button by using the other hand on a response box, with the function of the buttons counterbalanced across participants. We instructed participants to press the yes-button as fast as possible for learned (target) items, and the no-button for all other items. The task consists of a practice block (21 items) and two test blocks, each with 180 test items (3 target items repeated 10 times; 3 same identity items repeated 5 times; 3 other identity items repeated 5 times; 12 irrelevant items repeated 10 times, and one buffer item additionally at the beginning of each block). In total, there were 30 target trials, 15 other identity trials, 15 same identity trials, and 120 irrelevant trials per block; the proportion of relevant (same identity and other identity) items to irrelevant items thus was 1∶4, as is standard in the concealed information test. The test items appeared in random order without further constraints, with an equal number of items from each stimulus category presented in each block. Each word appeared in white capital letters on a black screen for 800ms. When no response was given within the 800 ms response deadline, feedback (“TOO SLOW” in red capital letters) was given for 1s. The inter-stimulus interval varied (either 500, 800, or 1000 ms). During the entire task a heading (DO YOU RECOGNIZE THIS WORD?) and the response labels (YES and NO presented on opposite sides of the screen) remained on the computer screen.

Patients as well as simulators performed the learning phase and the concealed information phase in their amnesic identity. We instructed simulators after the learning task. The rationale of the concealed information task was explained to the simulators and it was mentioned that stimuli pertaining both to the amnesic and trauma identity were included based on their answering in the autobiographical information questionnaires. We told them to try to hide recognition of the items pertaining to the trauma identity by not responding any faster or slower to the words pertaining to the trauma identity compared to irrelevant items.

## Results

One patient did not complete testing as she found the switching on demand too strenuous. Another patient did not report amnesia on the autobiographical questions pertaining to the other participating identity, and therefore was excluded in the analyses reported below. The data described below thus pertain to nine DID patients. These patients all indicated that switches during testing were successful and that other identities did not interfere with task performance. We excluded data for one simulator because debriefing indicated she had not understood the instructions. We report effect size r [30] for repeated measures ANOVA analyses and Coheńs d as a measure of effect size in post-hoc comparisons.

Demographics appear in Table 1. As the assumption of normality was violated, as indicated by significant Kolmogorov-Smirnov statistics, we present nonparametric Kruskal-Wallis tests and Mann-Whitney tests below. DID patients, controls, and simulators did not differ significantly on mean age, *χ^2^*(2) = .39, *p* = .82, and mean level of education, *χ^2^*(2) = 3.56, *p* = .17. The groups did differ on mean dissociation (as measured by the DES), *χ^2^*(2) = 23.62, *p*<.001, with Mann-Whitney tests indicating DID patients scored higher compared to controls, *Z* = 4.44, *p*<.001, and simulators, *Z* = 4.34, *p*<.001, whereas controls and simulators not differing significantly, *Z* = 1.23, *p* = .22. Groups also differed significantly on somatoform dissociation (as measured by the SDQ-5), *χ^2^*(2) = 27.52, *p*<.001, with Mann-Whitney tests indicating DID patients scored higher compared to controls, *Z* = 4.84, *p<*.001, and simulators, *Z* = 4.33, *p<*.001, and controls and simulators not differing significantly, *Z* = 1.23, *p = *.22.

### Personal Relevance Rating

Mean stimulus ratings for personal relevance are depicted in Table 2. As the assumption of normality was violated, as indicated by significant Kolmogorov-Smirnov statistics, we present nonparametric Wilcoxon signed ranks tests below. For DID patients and simulators, the ratings for other identity items were not significantly different compared to irrelevant items indicating the expected subjective amnesia (*Z = *1.66, *p = *.10, and *Z = *.13, *p = *.90, respectively). As expected, ratings for the same identity items were significantly higher as compared to the other identity items for all groups (*Z = *2.67, *p = *<.01 for patients; *Z = *4.21, *p*<.001 for simulators; *Z = *4.55, *p*<.001 for controls). Unexpectedly, controls rated the ‘other identity’ items slightly less personally relevant compared to irrelevant items, *Z = *2.49, *p = *.013.

### Concealed Information Task Results

Consistent with previous research [31], control subjects had very low overall error rates. Because controls did not show a significant difference on the critical comparison same identity items versus irrelevant items [*t* = 1.45, df = 26, *p* = .16], we regard the error scores as insufficiently sensitive to warrant further analysis. For sake of completeness we report the error rates in Table 3.

We calculated median reaction times (in ms) with classification errors excluded. Mean reaction time data for target, irrelevant, other identity and same identity items are depicted in Table 4. Individual difference scores are depicted in Figures 2 and 3. Non-significant Kolmogorov-Smirnov test results indicated normality for all reaction time data per diagnosis group. Box’s M statistic also was nonsignificant, indicating homogeneity of variance-covariance matrices. As Mauchly’s test of sphericity was significant, we report multivariate test results (Wilks’Lambda) below. A 4 (stimulus type: target, irrelevant, other identity, same identity) × 3 (group: DID patients, control participants, simulators) repeated measures ANOVA was conducted on the median reaction times. The results indicated a significant main effect of stimulus type, *F*_3,54_ = 226.58, *p*<.001 (*r* = .90). The effect of group was not significant, *F*_2,56_ = 2.70, *p = *.076 (*r = *.21), but the stimulus type × group interaction effect was, *F*_6,108_ = 2.69, *p* = .018 (*r = *.16). Crucial for the hypothesis of interidentity amnesia is the comparison other identity words versus irrelevant words in patients. The results indicate that DID patients took longer to classify other identity words than irrelevant words *t* = 2.87, df = 8, *p* = .021 (Cohen’s *d* = .45), whereas their RTs for other and same words were indistinguishable, *t* = 0.32, df = 8, *p = *.11 (*d* = .06), see Figure 4 (error bars indicate standard deviations). In striking contrast, for controls, classification RTs were indistinguishable for other identity words and irrelevant words, *t* = 0.67, df = 26, *p* = .51 (*d* = .08), whereas RTs were significantly slower for same identity words than other identity words, *t* = 3.99, df = 26, *p*<.001, (*d* = .69). For the simulators, RTs were significantly longer on other identity words compared to irrelevant words, *t* = 3.51, df = 22, *p* = .002 (*d* = .45), and also on same identity words compared to other identity words, *t* = 2.44, df = 22, *p* = .02 (*d* = .39). Confirming the sensitivity of the RT measure, all groups were slower to classify same identity words than irrelevant words (see Figure 4), *t* = 5.47, df = 26, *p*<.001 (*d* = 76) for controls; *t* = 1.86, df = 8, *p* = .10 (*d* = .50) for DID patients; *t* = 4.47, df = 22, p<.001 (*d* = .80) for simulators.

We found memory transfer of autobiographical information between identities in DID patients. Objective data fail to confirm subjective reports of amnesia in DID.

## Discussion

We assessed transfer of autobiographical information between identities in nine DID patients. Although the tested identity reported amnesia for material harbored by another identity, the RT data revealed recognition of the supposedly amnesic material. Our data indicate that the objective memory data fail to confirm subjective reports of amnesia in DID.

For control participants, RTs to same identity items were, as expected, significantly increased compared to RTs for irrelevant items (i.e., ‘other’ identity items and irrelevant items). The autobiographical information thus popped out, analogous to the so-called own name effect; that is, if someone calls out your name from across the room in a noisy place like a cocktail party, you will usually notice [32], [33]. These findings confirmed the sensitivity of our test. We expected the DID patients, in case of dissociative amnesia, to show comparable responses on other identity items and irrelevant items, reflecting the presumably impersonal nature of the other identity items. In case of transfer of information, we expected the DID patients to show responses on other identity items comparable to same identity performance (i.e., longer reaction times compared to irrelevant items). The main results thus indicate that, although patients subjectively reported amnesia for the selected autobiographical information reported by the other identity, the results on the concealed information task revealed transfer of information between identities**.** A limitation of the current study is the small sample size. However, the within-subject comparison design enabled satisfactory power levels.

Simulators received detailed instructions on how to simulate the expected amnesia response pattern (i.e., not responding any faster or slower to the other identity items compared to the irrelevant items). However, as they reacted significantly slower on other identity trials compared to irrelevant trials, they failed to mimic the exact profile as expected for amnesic DID patients. Simulation thus proved ineffective as they were unable to hide recognition of the other identity items. The debriefing indicated that the recognition of the other and same identity items increased reaction times as the simulators were deciding on the ‘right’ answer. For them, both the same identity *and* other identity items contain a response conflict, requiring a nonrecognized (“No”) response to a recognized item.

The personal relevance ratings indicated that for all participants, as expected, the same identity items were rated much higher on personal relevance compared to the other identity items, whereas for DID patients and simulators, the other identity items were rated comparably to irrelevant items. For control participants, the ‘other identity’ (consisting of irrelevant items) items were rated slightly, but significantly, less personally relevant compared to irrelevant items. This seems a chance finding. Importantly, the slightly higher ratings of the other identity items did not interfere with performance on the concealed information task, as control subjects did not show a differential error or reaction time pattern for other identity items compared to irrelevant items.

Our findings are consistent with the results of other studies involving objective laboratory tasks indicating intact inter-identity memory functioning in dissociative identity disorder. In most studies, researchers test memory within the same experimental session shortly after learning. In contrast, we tested memory after a 2-week delay, thus increasing the ecological validity of our study**.** However, the results do conflict with the reports of amnesia between identities, suggesting that the subjectively experienced absence of autobiographical knowledge about other identities is quite self-convincing.

One might argue that memory transfer between identities in our study reflects implicit rather than explicit memory [35]. However, in case of implicit memory transfer, we would have expected the DID patients to indicate some familiarity with the other identity items (i.e., compared to the irrelevant items) while rating the self-relevance of the stimuli. Also, debate persists regarding whether memory impairments in DID involve implicit memory in addition to explicit memory [36]. Cardeña [37], for example, stated that “even though conscious recollection may be absent, the information that cannot be recalled may still affect behavior (a deficit of explicit, but not of implicit, memory)” (p. 55) and “in dissociative amnesia, the individual loses explicit memory for personal experience, although implicit memory for general knowledge, skills, habits and conditioned responses is usually unimpaired” (p. 57). The *DSM-IV-TR* [1] defines amnesia in DID as the “inability to recall important personal information that is too extensive to be explained by ordinary forgetfulness” (p. 487), thus implying that only explicit memory is impaired [8], [38]–[39]. In contrast, Spiegel, Frischholz, and Spira [40] stated that amnesia between identities implies distinct memory storage structures that are functionally independent of one another. “Episodic memory developed by one personality is often not accessible by another. In many cases, even implicitly stores procedural memory is discrete” (p. 767; see also [41]). Putnam [42] mentioned that “fluctuations in the level of basic skills, in habits, and in recall of knowledge are classic forms of memory dysfunction in dissociative patients. Typically, dissociative patients describe suddenly ‘drawing a blank’ when asked to do something that they are familiar with. Paradoxically, it seems as if overlearned information and skills are especially susceptible to intermittent failures of memory retrieval” (pp. 82–83).

At the very least, our data are inconsistent with the definition of dissociative amnesia in DID as entailing separate inter-identity memory systems divided by impermeable amnesic barriers. The DID patients exhibited memory transfer across identities even though they did not realize it.
